# Pathophysiology and treatment of exercise-associated hyponatremia

**DOI:** 10.1007/s40618-025-02673-7

**Published:** 2025-09-06

**Authors:** Barbara Altieri, Irene Aini, Giuseppe Cannavale, Caterina Magnelli, Camilla Mancini, Virginia Zamponi, Andrea M. Isidori, Annamaria Colao, Antongiulio Faggiano, Alessandro Peri

**Affiliations:** 1 Division of Endocrinology and Diabetes, Department of Internal Medicine I, University Hospital, University of Würzburg, Würzburg, Germany; 2 Department of Medicine and Surgery, Division of Endocrinology and Metabolic Diseases, University of Parma, Parma, Italy; 3 Endocrinology Unit, Azienda Ospedaliera Universitaria Sassari, Sassari, Italy; 4 Endocrinology, Diabetology and Andrology Unit, Department of Clinical Medicine and Surgery, Federico II University of Naples, Naples, Italy; 5 Department of Experimental and Clinical Biomedical Sciences “Mario Serio”, University of Florence, Florence, Italy; 6 Endocrinology Unit, Department of Clinical and Molecular Medicine, Sant’Andrea University Hospital, ENETS Center of Excellence, Sapienza University of Rome, Rome, Italy; 7 Department of Molecular Medicine, Sapienza University of Rome, Rome, Italy; 8 Division of Endocrinology and Andrology, Department of Experimental Medicine, Sapienza University, Rome, Italy; 9 UNESCO Chair Education for Health and Sustainable Development, Federico II University, Naples, Italy; 10 Pituitary Diseases and Sodium Alterations Unit, Endocrinology, Careggi University Hospital, Florence, 50139 Italy

**Keywords:** Hyponatremia, Exercise-associated hyponatremia, Overhydration, Physical exertion, Vasopressin

## Abstract

Exercise associated hyponatremia (EAH) is a medical condition that can occur during physical exertion. Initially, EAH was considered to be restricted to extreme endurance activities, such as ultramarathons and Ironman triathlons. However, it has been more recently recognized in a variety of sports, including team sports and in shorter-duration events. The pathophysiology of EAH is multifactorial and includes excessive fluid intake and non-osmotic arginine vasopressin secretion, which is induced by physical activity. Sodium loss through sweat appears to play a less important role in contributing to EAH. The clinical presentation may vary, depending on the degree of serum sodium reduction. Symptoms, which are due to increased intracranial pressure, may vary from nausea, vomiting, headache, confusion to severe alterations in cognitive functions, decorticate posturing, respiratory distress, coma and even death. It is of pivotal importance to differentiate EAH from other conditions that may present with similar signs/symptoms, such as for instance hypoglycemia, orthostatic hypotension, vasovagal syncope, heat stroke. The treatment of EAH depends on the severity of symptoms. In life-threatening situations intravenous infusion of hypertonic saline solution (3%NaCl) is recommended. In less severe situations oral hypertonic saline solutions can be administered, as an alternative to intravenous hypertonic saline, when tolerated by patients. When symptoms are negligible, the treatment can be limited to fluid restriction. Effective strategies to prevent EAH would be important to reduce the risk of incurring in potentially life-threatening situations. In particular, recommendations to drink in anticipation of thirst during physical exertions should be replaced by the “drinking when thirsty” strategy.

## Introduction

Exercise-associated hyponatremia (EAH) represents a critical yet frequently underappreciated medical condition in sports medicine. Despite its potentially life-threatening consequences, it remains overshadowed by other exercise-related metabolic disturbances. EAH occurs when serum sodium levels drop below the physiological range, a condition commonly observed during prolonged physical exertion, particularly in endurance events [1]. The pathophysiology of EAH involves a spectrum of causes, from excessive fluid intake to impaired renal excretion due to non-osmotic arginine vasopressin (AVP) secretion, which is induced by exercise [2–5]. The clinical presentation of EAH can range from mild symptoms such as bloating and nausea to more severe and life-threatening conditions like encephalopathy, cerebral edema, and pulmonary edema [6, 7].

Initially considered rare and confined to extreme endurance activities like ultramarathons and Ironman triathlons (a long-distance triathlon which consists of a 3.9 km swim, a 180.2 km bicycle ride and a marathon 42.2 km run), EAH has since been recognized in a variety of sports, including team sports and even shorter-duration events [8–12]. This growing recognition reveals the complexity of the condition, which is frequently underestimated by athletes, coaches, and medical professionals. Risk factors for developing EAH include overdrinking, prolonged endurance activity, event inexperience, and warmer environmental conditions [6, 13].

The prevention of EAH remains poorly understood and inadequately addressed. Despite mounting evidence that fluid intake should be guided by thirst to avoid hyponatremia, outdated hydration guidelines and the aggressive marketing of sports drinks continue to promote excessive fluid consumption [8, 14]. Many athletes are still encouraged to drink beyond physiological needs, a practice that increases the risk of developing EAH [15, 16].

As EAH becomes increasingly recognized in a wide variety of sports, there is a pressing need for further research to better understand its pathophysiology, especially regarding the balance between fluid intake, sodium loss through sweat, and the role of non-osmotic AVP secretion. This review seeks to provide a comprehensive analysis of the incidence, risk factors, diagnosis, treatment, and prevention of EAH, emphasizing the need for heightened awareness of this under-researched and often underestimated medical issue within sports. By doing so, it aims to encourage a more informed approach to hydration strategies in athletic contexts, potentially reducing the incidence of this preventable yet serious condition.

## Epidemiology

EAH is often discovered incidentally in athletes who show no symptoms through routine screenings. An incidence of asymptomatic EAH ranging between 5 and 70% in athletes after endurance events has been reported [5, 12, 17–23]. Symptomatic EAH is less frequent and occurs in 0.1% to 1.0% of endurance athletes, with marathoners, ultramarathoners, Ironman competitors, long-distance hikers, and military personnel being the most frequently affected [17, 18, 20, 24–26]. A few cases of severe symptomatic EAH followed by death have been reported, including football players [27, 28], an ironman triathlete [29], a canoeist during a 120-mile race [30], and soldiers [31, 32]

The primary risk factors for EAH include overhydration, environmental conditions and exposure to high temperatures, and prolonged physical activity, typically lasting more than 2 h [33–35] Particularly, numerous studies showed a direct correlation between temperature and the occurrence of EAH [6, 24]. In marathon runners it has been shown that participants who gained weight during the race—a marker of overhydration—were more likely to develop EAH than those who lost weight [19, 33]. It has been suggested that menstruating women may be at a higher risk of developing EAH compared to men [7, 33], potentially due to estrogen’s role in hindering the brain’s ability to adapt to rapid osmotic swelling [36]. However, a large-scale study of Boston Marathon participants indicated that the observed sex difference vanished when the data were adjusted for body mass index and race times [11].

## Etiopathogenesis

In EAH fluid overload may be shown not only by changes in electrolyte levels, but also by alterations in plasma volume [37, 38], and albumin concentration [39]. The etiopathogenesis of EAH involves a complex interplay of factors with multiple overlapping etiologies, including excessive fluid intake, inappropriate AVP secretion, sodium loss, and individual susceptibility [40].

The primary cause of EAH is the overconsumption of fluids, which dilutes the sodium concentration in the blood [40, 41]. Athletes often drink large amounts of water or other hypotonic fluids during and after prolonged exercise, driven by the belief that more fluid intake enhances performance and prevents dehydration [1, 42]. This overhydration can lead to a state where the body’s capacity to excrete the excess water, by sweating, urine and insensible losses (mainly respiratory and gastrointestinal), is overwhelmed, resulting in dilutional hyponatremia. Studies have shown that athletes who consume fluids beyond the dictates of thirst are at a significantly higher risk of developing EAH. For instance, research on marathon runners has indicated that those who gained weight during the race, indicative of fluid overload, had a higher prevalence of EAH compared to those who maintained or lost weight [28, 41].

The inappropriate secretion of AVP, also known as anti-diuretic hormone, is another critical factor in the pathogenesis of EAH. AVP promotes water reabsorption in the kidneys, reducing urine output and contributing to fluid retention. During prolonged exercise, AVP secretion can be stimulated by non-osmotic factors such as physical stress, pain, nausea, and hypoglycemia, leading to water retention despite a hypotonic plasma state. The secretion of AVP during endurance activities is thought to be a protective mechanism aimed at preserving blood volume and maintaining cardiovascular stability. However, in the context of excessive fluid intake, this mechanism can become maladaptive, exacerbating the dilution of serum sodium [2–4, 43, 44]. An additional mechanism could be the induction of AVP secretion by the release of inflammatory cytokines, particularly interleukin-6 (IL-6). IL-6 increases during exercise due to its role in energy mobilization and muscle inflammation from breakdown. A study on 15 ultramarathon participants found a significant positive correlation between AVP and IL-6 but no correlation was observed between AVP and blood glucose or ambient temperature [5].

Sodium loss through sweat is an additional factor that possibly contributes to EAH, particularly in endurance athletes who exercise for extended periods. While the sodium concentration in sweat is highly variable between individuals, and endurance athletes generally have lower sodium levels in their sweat compared to the general population, their total sodium loss can still be considerable. This is because endurance athletes tend to sweat more due to the prolonged duration and intensity of their exercise. Therefore, even with a lower sodium concentration per unit of sweat, the increased sweat volume leads to a greater overall sodium loss. If this sodium is not adequately replaced, in principle it can contribute to the development of hyponatremia [45, 46]. However, there are studies that showed inconsistent effects of sodium supplementation in preventing EAH [47–49]. Accordingly, the role of sodium loss in contributing to the etiopathogenesis of EAH does not appear supported by the observation that in ultramarathon runners that developed hyponatremia a markedly higher fluid retention, yet not different sodium loss, was observed compared to runners that remained normonatremic [50].

Another aspect to be considered is that a significant portion of the body’s sodium is stored in bone, skin, and cartilage, bound to substances like glycosaminoglycans, and can shift between osmotically active and inactive forms [51]. This dynamic pool may account for up to a quarter of the body’s total sodium [52]. Sodium from this pool may help to prevent hyponatremia or, conversely, worsen it. In athletes with EAH, the inability to activate this sodium pool or a shift to inactive forms could explain the condition [53]. Though the mechanisms are unclear, this theory might explain why some athletes gain weight without becoming hyponatremic.

In addition to excessive water intake, glycogen metabolism may contribute to hyponatremia without weight gain, as metabolizing glycogen releases water that can dilute serum sodium. Endurance exercise also activates neurohormonal systems like the sympathetic nervous system and renin–angiotensin–aldosterone system, which increase sodium and water reabsorption, impairing free water excretion. Post-event, rapid fluid absorption from the gastrointestinal tract can exacerbate hyponatremia [40].

According to various case reports, EAH might induce rhabdomyolysis [54–57]. Rhabdomyolysis can result from osmotic shock of skeletal muscle membrane, the activation of reactive oxygen, and increased intracellular calcium, which activate proteases and cause cellular breakdown [58]. This process triggers local and systemic inflammation, thus inducing the release of IL-6, which may elevate AVP levels, further lowering serum sodium [43, 54].

## Clinical manifestations and diagnosis

The clinical manifestations of EAH are vague and include a spectrum of signs and symptoms that are common to other conditions potentially induced by physical exertion. Therefore, it is crucial to carry out an accurate differential diagnosis from all conditions that may present with similar signs/symptoms, such as hypoglycemia, orthostatic hypotension, hypovolemia, vasovagal syncope, hypernatremia, heat stroke, heat exhaustion, and sudden cardiac arrhythmia.

**Fig. 1:**
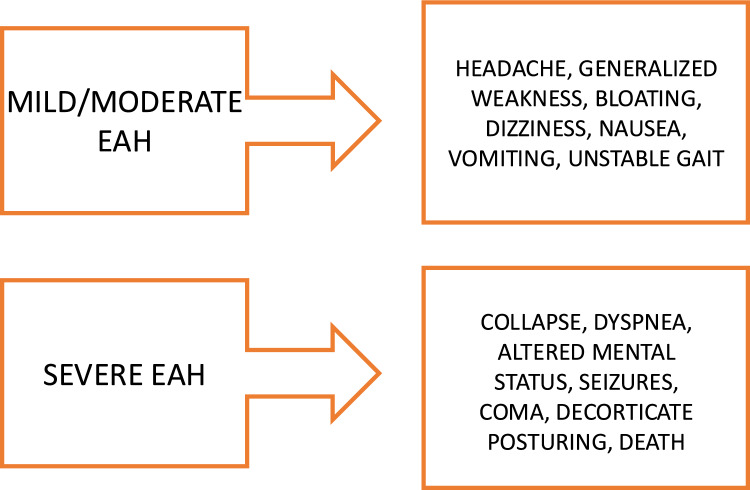
Signs and symptoms in mild/moderate and severe exercise-associated hyponatremia (EAH)

The search for signs/symptoms of hyponatremia and the assessment of their severity is essential to implement the most appropriate therapeutic approach [6, 26]. EAH is asymptomatic in most cases and low serum sodium levels are often found during examinations conducted for research purposes. In contrast, symptomatic EAH is rare, occurring for instance in less than 1% of marathon runners [17, 59, 60]. Nevertheless, if we consider for instance that more than 55,000 runners participated to the 2025 New York City marathon, an estimated number of about 550 cases of EAH is not negligible. Symptomatic EAH can present with varying degrees of severity and can be distinguished into a mild and a severe form (Fig. 1), which require different therapeutic approaches. The finding of serum sodium levels below 135 mmol/L associated with a positive history of excessive consumption of hypotonic fluids or sports drinks allows for an easier diagnosis of EAH. The suspicion of EAH should be strong in the presence of altered mental status, bloating, and absence of thirst. Otherwise, signs of dehydration, thirst, orthostatic hypotension, and elevated core temperature should steer the diagnostic hypothesis toward heat disease [17, 26, 59]. The most common symptoms in mild/moderate EAH are bloating, vomiting, nausea, headache and altered mental status. Nearly all symptoms are neurological and are due to increased intracranial pressure from cellular edema. The most severe expression of EAH includes significant alterations in cognitive functions such as confusion, coma, decorticate posturing, and respiratory distress due to the onset of cerebral (exercise-associated hyponatremic encephalopathy, EAHE) and/or pulmonary edema [6, 17, 26, 59–61], possibly leading to death.

The initial assessment involves implementing the ABCDE protocol (airways, breathing, circulation, disability, exposure), which includes measuring vital signs, determining the level of consciousness, maintaining airway patency, and supporting circulation. Additionally, it is crucial to assess rectal temperature and blood glucose levels to exclude other potential causes with similar signs/symptoms, as well as to conduct an accurate interview with the patient or a witness to investigate the extent of physical exertion and the environmental conditions in which it occurred [26, 59, 62].

Determining volume status in athletes with EAH is often challenging, but when feasible, it allows for the distinction of EAH based on the extracellular volume, which can be normal, increased or reduced. Weight gain, reduced blood urea nitrogen (BUN), and urinary sodium levels above 30 mmol/L suggest the presence of increased or normal extracellular volume, whereas weight loss, increased BUN, and urinary sodium levels below 30 mmol/L [6] are indicative of reduced extracellular volume.

## Therapy

Hyponatremia secondary to exercise is almost always acute, which is defined as a hyponatremia occurred in the last 48 h. The presence of dysnatremia negatively impacts the speed of recovery of athletes involved in long or demanding races such as marathons or endurance races. In a 2007 study of athletes who collapsed during the 2005 Comrades Marathon, normonatriemic athletes spent less time in the medical tent (80 ± 31 min) compared with hypernatremic (102 ± 36 min) and hyponatremic (146 ± 122 min) runners [63]. Therefore, to optimize the treatment of athletes requiring assistance, sodium levels should be tested using point-of-cares [64].

**Fig. 2:**
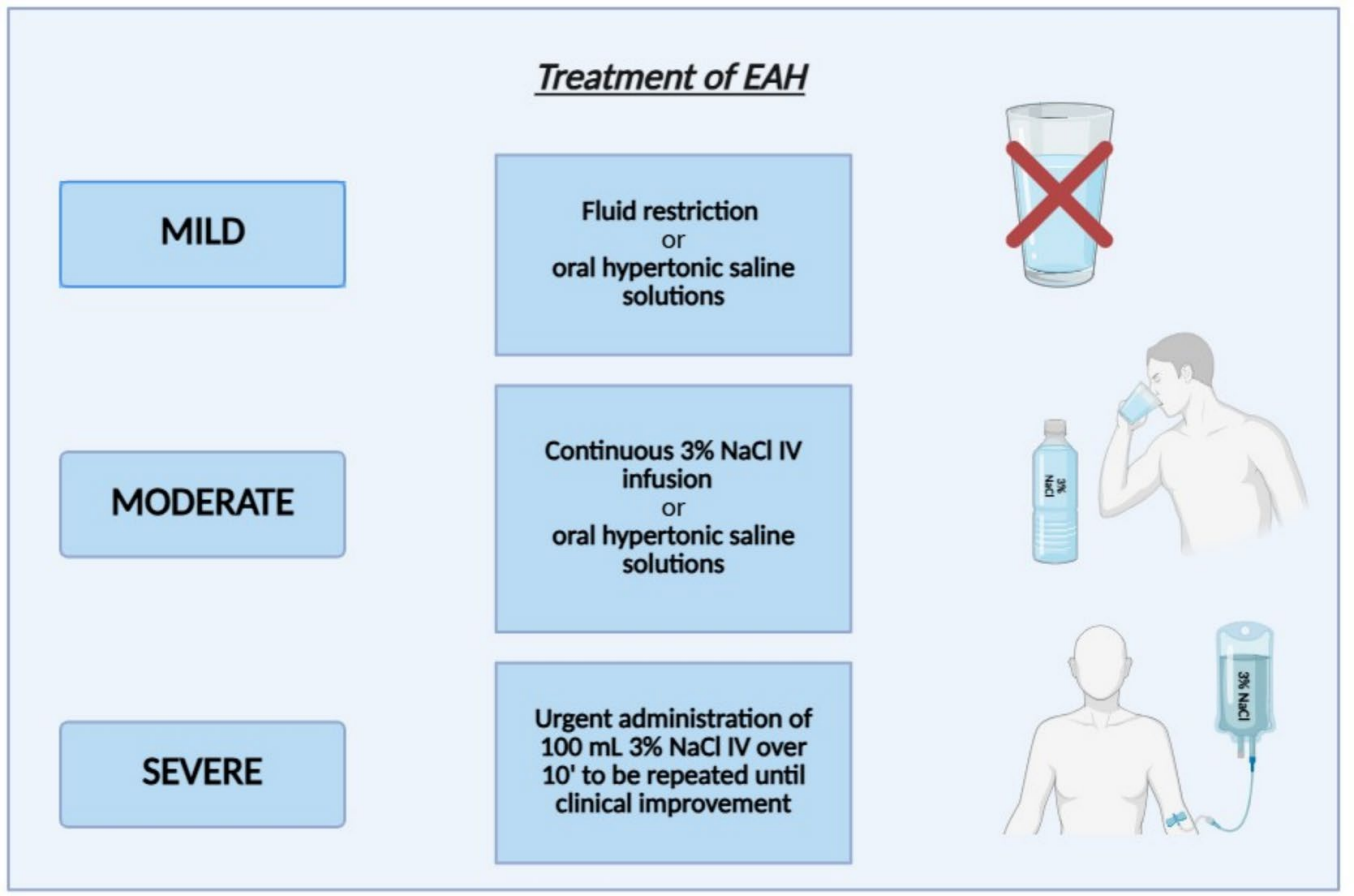
Treatment strategies for mild, moderate and severe exercise-associated hyponatremia (EAH)

The treatment of EAH is guided by the presence of signs and symptoms related to serum sodium levels, according to other causes of acute hyponatremia. Severe forms of EAH are characterized by the onset of encephalopathy sometimes also associated with pulmonary edema. They are life-threatening forms due to possible brain herniation, thus requiring acute treatment with hypertonic saline (3%NaCl) intravenously to be repeated up to three times until improvement of symptoms [6]. Mild-to-moderate forms, characterized by a wide spectrum of symptoms and signs, which may vary from almost negligible ones to nausea, vomiting, headache, and possible moderate mental status changes, can be treated with fluid restriction (milder forms) or with oral hypertonic saline solutions or continuous 3%NaCl IV infusion [6], when clinical features are more evident (Fig. 2).

In a 2014 study of athletes participating in the 161-km Western States Endurance Run out of 48 hyponatremic patients 16 were treated immediately with intravenous hypertonic saline solution (3%NaCl) for critical conditions, the remainder were treated with either oral or intravenous therapy (3% NaCl). Both treatments were effective at one hour with an average increase in blood Na of about 2 mmol/L (p < 0.0001). However, no significant differences were found between the group treated with 3% intravenous hypertonic solution (serum sodium from 129.8 ± 4.2 mmol/L to 131.8 ± 3.8 mmol/L) and the group treated with oral hypertonic solution (serum sodium from 131.5 ± 2.1 mmol/L to 133.4 ± 2.3 mmol/L) [65]. A 2020 study of participants in the annual long-distance triathlon (3.8-km swim, 180-km bike, and 42-km run) at Mont-Tremblant, Canada also showed no differences in the treatment efficacy of mild-to-moderate forms of hyponatriemia between intravenous hypertonic solution (3% Na Cl) and oral solution. Surprisingly, patients treated with oral solutions showed a more rapid recovery (50.3 min vs. 75.8 min). Discharge criteria were resolution of symptoms, autonomy in walking and urinate freely on their own [24].

In principle, in acute hyponatremia, which is the situation that almost invariably occurs in EAH, the risk of developing osmotic demyelination syndrome, secondary to an overly rapid correction of serum sodium, is low [66]. Yet, serum sodium levels need to be monitored. With regard to this point, whereas the U.S. recommendations for the management of hyponatremia did not established a limit for serum sodium correction in acute hyponatremia, the European guidelines recommended a correction limit of 10 mmol/L in the first 24 h of treatment in all hyponatremic patients, thus including acute hyponatremia [67, 68].

## Prevention

Given that the predominant pathogenetic mechanism of EAH is plasma dilution due to both excessive fluid reintroduction (overdrinking) and altered AVP-mediated renal clearance of free water, the main, and safest, individual preventive strategy against EAH is to drink according to thirst; before, during and immediately after exercise [1].

Establishing universally how much fluid to take in is not possible as there is a wide variability in fluid elimination by sweating and renal excretion both between different athletes in the same environment and in the same athlete under different environmental conditions [69]. This strategy -drinking when thirsty- makes it possible to replace losses from sweating without incurring in overcorrection, as demonstrated in a prospective study conducted on marathon runners [70], and thus prevent both dilution-related EAH and reduced performance from excessive dehydration in most cases [71]. Some genetic or local factors (such as xerostomia) could be exceptions in the success of this preventive strategy [72, 73].

The thirst mechanism is an evolutionarily conserved and finely tuned regulatory mechanism aimed at keeping plasma osmolality and circulating plasma volume constant in certain ranges [74]. Cerebral osmoreceptors and baroreceptors located in the aortic arch, carotid sinuses and large veins provide real-time input to higher brain centers that constantly coordinate the sense of thirst and excretion of AVP, urging us to drink when necessary. Recent evidence has also shown that certain brain areas (cingulate cortex, insula, amygdala and periaqueductal grey) are activated following the intake of an excessive amount of fluid compared to the sense of thirst, triggering an unpleasant sensation [75, 76]. Earlier recommendations to drink in anticipation of thirst were largely aimed at situations where sweating rates were high, greater than the rate of gastric emptying, and rapidly harbingers of dehydration; however, the result of this strategy has unfortunately fostered the misconception that thirst is not a reliable signal in replenishing lost fluids and has facilitated overdrinking and thus the development of EAH [1]. Nonetheless, there is no evidence to support this idea: overdrinking has not been shown to decrease fatigue, muscle cramps or exertional heat stroke and, moreover, modest levels of dehydration (approximately 3% of normal body mass or 5% of total body water content) may be well tolerated [1, 8].

To limit access to water intake, it is useful to distance the fluid stations further apart along the course. In the 2002 Christchurch Marathon, in addition to discouraging over-hydration, fewer refreshment stations were placed than in the Boston Marathon of the same year (every 5 km versus 1.6 km): in the 134 marathon runners who finished the course in the first city, there were no cases of hyponatremia compared to a 13% incidence at the end of the Boston Marathon [10, 11]. Similarly, in the case of Ironman it is recommended, in association with adequate rehydration education, to place refreshment stations every 2.5 km along the running route and every 20 km along the cycling route [1, 77].

Although the American College of Sports Medicine (ACSM) recommends the intake of 500–700 mg (22 to 30 mEq) of sodium per liter of water as an appropriate dose to restore sodium losses through sweating during endurance activities [78], there is to date insufficient evidence to show that such supplementation is able to prevent or reduce the risk of EAH, similarly for the intake of the so called “sports drinks”, whose main components are carbohydrates and/or electrolytes [1, 79, 80]. These are mostly hypotonic drinks containing a markedly lower concentration of sodium than the serum concentration (10–38 mmol/L versus 140 mmol/L): the dilution resulting from the volume of excess fluid introduced (overdrinking) cancels out and overrides the effect of the sodium and other electrolytes contained in such sports drinks [81]. As demonstrated in one study [49], in which 376 participants in a 161 km endurance race were subjected to a questionnaire on the type of drink consumed during the ultramarathon and to post-race natremia assessment blood samples, sodium supplementation is not necessary for the prevention of EAH, whether in prolonged activities such as an ultramarathon (duration of more than 18 h) or those of shorter duration [82]. On the contrary, it can sometimes result in adverse events arising from the excessive water retention it induces [49, 82]. Although in some studies [79] the plasma sodium concentration increased following oral supplementation, this increase was not found to be statistically significant compared to the control group and, in general, in those who consume 1.5 g of sodium daily in the diet, as recommended by the Institute of Medicine (USA), this dose is sufficient to restore salt losses that occur through sweating during physical activity [9, 83].

The use of the USA Track and Field (USATF) guidelines, or other similar methods, to estimate hourly sweat losses during exercise and thus avoid excessive fluid consumption during endurance sport events could theoretically be employed and facilitated through serious body weight measurements taken both during and after exercise with the aim of maintaining the initial weight or achieving a slight reduction in it [84]. However, the results obtained during a training session may not be replicated during a competition when AVP secretion may not be suppressed and, in addition, these guidelines would be more difficult to follow for occasional athletes who are at greater risk of hyponatremia (especially if the duration of exercise is longer than 4 h, as demonstrated in the 2002 Boston Marathon) [11]. Furthermore, during exercise the body’s regulatory mechanisms tend to safeguard plasma sodium concentration, not body weight, the variation of which would in any case be unrepresentative of changes in total body water content; in fact, the main information it can give us is possibly fluid overload if it is increased [1, 85]. The strategy of drinking in accordance with thirst, however, seems to provide sufficient protection [1, 17, 86].

Finally, the prevention of EAH might be obtained above all through the development of educational programmes that emphasise the importance of good hydration practices, educate on the symptoms of EAH for early recognition and appropriate treatment. In a study conducted among London Marathon participants, it was found that 68% of them were aware of the possibility of developing hyponatremia, 35.5% were actually aware of its causes and consequences, and only 25.3% of the participants planned to hydrate according to their thirst during the marathon, whereas 12% planned to take in a volume of fluids that would put them at risk of developing EAH [9, 87]. The dissemination of position statements released by sport authorities among athletes should be also encouraged [88, 89]. Furthermore, advice given to participating athletes should always be reviewed and approved by the event’s medical team, which should ideally also have Point Of Care Testing available for on-site assessment of plasma sodium to diagnose and manage EAH early [1].

## Conclusions

This review extensively described EAH, a medical condition that can occur during physical exertion and that is not limited to extreme endurance activities, as it was initially thought. Multiple factors can induce EAH and among them a crucial role is played by excessive fluid intake, which derives from earlier recommendation to drink in anticipation of thirst. EAH may be a life-threatening condition, which requires prompt medical intervention to increase serum sodium and reduce cerebral edema. Of course, preventing EAH remains the main goal to achieve. Therefore, effective strategies should be reinforced, through the development of educational programs, aiming to increase for instance the awareness of the importance of good hydration practices. To achieve this goal, national sports authorities, as well as local sports societies, should play a pivotal role in promoting and supporting information campaigns.

## Data Availability

No datasets were generated or analysed during the current study.
